# A practical guide to in situ and ex situ characterisation of arabinogalactan proteins (AGPs) in fruits

**DOI:** 10.1186/s13007-023-01100-3

**Published:** 2023-11-01

**Authors:** Nataliia Kutyrieva-Nowak, Agata Leszczuk, Artur Zdunek

**Affiliations:** Institute of Agrophysics, Polish Academy of Sciences, Doświadczalna 4, 20-290 Lublin, Poland

**Keywords:** Arabinogalactan proteins, Cell wall, Glycobiology, Immunocytochemistry, Microscopy, Molecular biology

## Abstract

**Background:**

Arabinogalactan proteins (AGPs) are plant cell components found in the extracellular matrix that play crucial roles in fruit growth and development. AGPs demonstrate structural diversity due to the presence of a protein domain and an expanded carbohydrate moiety. Considering their molecular structure, the modification of glycosylation is a primary factor contributing to the functional variety of AGPs.

**Main body:**

Immunocytochemical methods are used for qualitative and quantitative analyses of AGPs in fruit tissues. These include in situ techniques such as immunofluorescence and immunogold labelling for visualising AGP distribution at different cellular levels and ex situ methods such as Western blotting and enzyme-linked immunoenzymatic assays (ELISA) for molecular characterisation and quantitative detection of isolated AGPs. The presented techniques were modified by considering the structure of AGPs and the changes that occur in fruit tissues during the development and ripening processes. These methods are based on antibodies that recognise carbohydrate chains, which are the only commercially available highly AGP-specific tools. These probes recognise AGP epitopes and identify structural modifications and changes in spatio-temporal distribution, shedding light on their functions in fruit.

**Conclusion:**

This paper provides a concise overview of AGP research methods, emphasising their use in fruit tissue analysis and demonstrating the accessibility gaps in other tools used in such research (e.g. antibodies against protein moieties). It underscores fruit tissue as a valuable source of AGPs and emphasises the potential for future research to understand of AGP synthesis, degradation, and their roles in various physiological processes. Moreover, the application of advanced probes for AGP visualisation is a milestone in obtaining more detailed insights into the localisation and function of these proteins within fruit.

## Background

Arabinogalactan proteins (AGPs) are widely distributed components of the plant cell, where they serve a variety of functions. In general, AGPs have been found in all kinds of tissues, mostly in plasma membranes (PM), cell walls, and intercellular spaces as well as in soluble exudates secreted by plants. Studies at the cellular level have shown that the specific localisation of AGPs facilitates the formation of a continuum between the plasma membrane and the cell wall [1, 2]. AGPs are classified as proteoglycans with several combinations of glycosylated variations (glycoforms) [3, 4]. The AGP polypeptide sequence and the complexity of AG polysaccharide chains (presence of various sugars in varying amounts) are major factors that contribute to the high structural variability of AGPs [2, 5]. About 10% of the total molecular mass of proteoglycans is made up by the protein moiety [2]. As part of the hydroxyproline-rich glycoprotein family (HRGPs), AGPs contain a large number of hydroxyproline residues [2, 6–8]. Proline/hydroxyproline, alanine, serine, and threonine are also prevalent in their *N*-terminal domain [9–11]. During the biosynthesis and the post-translational modification of AGPs, the enzyme prolyl 4-hydroxylase (P4H) transforms proline (Pro) into hydroxyproline (Hyp), which is necessary for the molecule glycosylation process. AGPs are not properly glycosylated when proline hydroxylation does not occur, and changes in the proline hydroxylation process cause either their breakdown or a shift to lower molecular weight polypeptides [9, 12]. In turn, about 90% of the molecule consists of carbohydrate chains which are rich in arabinose, galactose, and occasionally D-glucuronic acid (GlcA), l-rhamnose (l-Rha), and uronic acids [13]. Most AGPs have one or more hydroxyproline residues that have been* O*-glycosylated by AG type II. They consist mostly of (1 → 3)-β-galactan and (1 → 6)-β-linked galactan chains attached to each other by (1 → 3, 1 → 6)-linked branch points with terminal arabinosyl residues in the *O*-3 and *O*-6 positions. The size of type II AGs varies between AGPs, with estimations typically lying between 30 and 150 sugar residues. The side chains of AGPs vary greatly due to the inclusion of different sugar residues in their structure. l-arabinose (l-Ara) residues are present in the side chains, along with GlcA, Rha, 4-*O*-methyl-glucuronic acid (4-Me-GlcA), d-xylose (Xyl), d-mannose (Man), d-glucose (Glc), d-galacturonic acid (GalA), d-glucosamine (GlcN), and l-fucose (l-Fuc) [4, 13, 14]. Both moieties allow the formation of an AGP molecule ranging in molecular weight from 60 to 300 kDa [15].

Moreover, there is a structure–function relationship in AGPs. The functionality of AGP is based on the direct function of its glycan [15]. It is known that AGPs have been related to various important stages of plant growth and development, including seed germination, somatic embryogenesis, pollen tube formation, cell division, cellular communication, and programmed cell death [4, 16]. AGPs have reportedly been shown as regulators of cell growth and differentiation processes, transducers of cell surface signals, and signalling pathways of responses to the environment [17, 18]. In situ and ex situ studies on AGPs in fruits performed by Leszczuk and coworkers have shown that AGPs are consistently found in fruit tissue and may induce alterations in fruits during the developmental and ripening processes [12, 19]. Analyses of AGP in fruits at the molecular level, i.e. ion binding, the establishment of cell wall-plasma membrane integrity, and cross-linking with other cell wall constituents [20], show that AGPs have an impact on fruit cell wall dissolution and subsequent softening [14].

Therefore, the characterisation of AGPs, particularly in terms of their hypothetical functions in fruit metabolism, necessitates the employment of numerous techniques, such as immunocytochemical methods based on the detection of carbohydrate epitopes. We have compiled a practical guide that includes methods that can be applied in AGPs research in fruits. The aim of the current paper is to provide step-by-step instructions for using and adapting common research techniques in studies on AGPs in fruits with the proviso that these methods should be correctly adjusted and modified before studying the specificity of AGPs in fruit tissue during the development and ripening processes. Moreover, this guide will equip researchers with tools with shown critical gaps which will make it easier to carry out analyses of fruit tissues and will also allow the potential of analyses of AGPs to understand the structure and changes of the cell wall during physiological processes.

## Immunocytochemical methods for qualitative and quantitative analyses of AGPs

AGPs can be found, measured, and localised using a variety of in situ and ex situ techniques [10]. However, due to the structural and compositional diversity of both the protein and carbohydrate moieties of AGPs, each technique has some advantages and disadvantages. Also, it is well known that fruit tissue is specific and requires appropriate treatment and selection of adequate methods [21, 22]. In situ research allows the study of biological processes in the context of the actual plant/fruit environment, which is important for understanding the AGP functions and interactions between individual extracellular matrix components as well as their response to all stress factors. However, in situ analyses must be complemented by equivalent ex situ studies. Ex situ studies of isolated AGPs may not accurately reflect processes occurring in the natural environment. The extraction process is always associated with the need to detach AGP molecules from the matrix, and the progress of the degradation process, especially of the protein moiety. Above all, only the analysis of isolated AGP makes it possible to describe the features of AGP that are the result of ongoing processes related to the cell functioning. Currently, one of the most effective approaches for studying AGPs is immunocytochemistry. Ex situ methods like immunoblotting, immunoprinting on the membrane, and enzyme-linked immunosorbent assay (ELISA test), which include molecular characterisation and quantitative detection, can be used to identify AGP epitopes. In turn, in situ methods used for this purpose are typically based on the immunofluorescence technique and immunogold labelling, allowing visualisation of the AGP distribution at the cellular and subcellular levels [23]. Both types of techniques facilitate a comprehensive description of the presence of AGPs in fruits.

The methods mentioned above are based on individual antibodies specifically recognising the target of interest. The antibody detects and binds to precisely defined antigen epitopes. By using particular monoclonal antibodies (mAbs) that bind to the structurally complex motif present in these proteoglycans, AGPs can be detected in plant tissues [24]. Moreover, the knowledge of the AGP glycan structure obtained via the widespread use of anti-AGP mAbs, such as JIM8, JIM13, JIM14, LM2, and others, demonstrates that AGPs are variably expressed during fruit growth and ripening [13]. Characterisation of commercially available antibodies commonly used in AGP studies is presented in Table 1.

**Table 1:** Characterisation of AGP epitopes recognized by specific antibodies

Antibody name	Epitope structure	Characterisation	M.W. of Protein Antigen (kDa)	References
JIM4	β-GlcA(1 → 3)-α-GalA(1 → 2)-Rha	Recognises oligosaccharides; binds to a set of discreet protein bands	70–100	[25–27]
JIM8	Carbohydrate portion of arabinogalactan proteins	Binds to a galactose-rich epitope of agps; the epitope contains one or more galactose residues	68, 84, 160	[25, 28, 29]
JIM13	β-GlcA(1 → 3)-α-GalA(1 → 2)-α-Rha	It is most likely not the complete epitope structure, because it binds to other anti-AGP glycan antibodies	80–100	[25–27]
JIM14	β-Gal(1 → 6)-β-Gal(1 → 6)-β-Gal(1 → 6)	Binds to at least three consecutive galactans with β-1,6-linked galactan; is used to visualise differentially branched galactan in type II AG polysaccharides	80–100	[25–27, 30]
JIM15	D-GlcA, GlcA-β(1-O-Me)	Recognises an epitope distinct from JIM13 and JIM14 but uncharacterised	80–100	[25–27]
JIM16	β-Gal(1 → 3)-β-Gal(1 → 3)-β- Gal(1 → 3)	Binds to a β-1,3-linked galactan backbone when substituted with a single β-1,6-linked Gal residue; used to visualise differentially branched galactan in type II AG	80–100	[25, 26, 30]
JIM101	unknown	Binds strongly to okra rhamnogalacturonan I and seed mucilages from *Sinapus alba* and *Camelina sativa*	No information	[31]
JIM133	β-1,3-linked galactooligosaccharides	Detects the nonreducing ends of the β-1,3-linked galactan backbone in the AG structures of agps	No information	[30, 31]
MAC204	Arabinogalactan (epitope structure unknown)	Binds to arabinogalactan proteins, but the epitope has not been characterised in detail	90–100	[31, 32]
MAC207	β-GlcA(1 → 3)-α-GalA(1 → 2)-Rha	Recognises an arabinose-containing epitope	70–100	[27, 29, 31, 32]
LM2	β-D-GlcA	Recognises a carbohydrate epitope containing β-linked D-glucuronic acid in AGP glycan	No information	[26, 28, 33]
LM14	AG type II arabinogalactan	Recognises arabinose and galactose-rich epitopes	No information	[34]
LM30	Arabinogalactan	Binds to terminal arabinose residues linked to galactan	No information	[35]
PN 16.4B4	Uncharacterised epitope in the carbohydrate part of the glycoprotein	Binds to arabinogalactan glycoproteins	135–180	[31, 36]

All immunocytochemical techniques are based on a general pattern consisting of sequential steps: material preparation (1), immunocytochemical reaction with primary and secondary antibodies (2), and signal detection and measurement using adequate tools for particular methods (3). First, a properly prepared fruit tissue is subjected to post-fixation membrane-permeabilisation. Although antigen–antibody binding is characterised by high specificity, there is a possibility of non-specific antibody interactions. To prevent and reduce nonspecific background staining, the blocking step is necessary before the immunocytochemical reaction, which involves incubation of the material with immunologically inactive (containing no specific antibodies) serum from another animal species, i.e. 2–10% solution of bovine serum albumin (BSA) [37]. In the next step, the examined material is incubated with an unlabelled antibody that recognises the specific antigen (primary antibody). Then, the excess antibody that did not bind to the antigen is washed off and a second incubation step is performed with a so-called secondary antibody conjugated with label molecules, which facilitates detection with well-established methods [38]. Here, protocols of the immunofluorescence labelling imaged with a confocal laser scanning microscope (CLSM), immunogold labelling imaged with the transmission electron microscope (TEM), immunoblotting, and ELISA test are described in detail with an emphasis on essential steps that should be modified in fruit AGP analyses.

### In situ studies—microscopic methods

Modern bioimaging methods facilitate the visualisation of epitope distribution at cellular and subcellular levels [39–41]. The presence of AGPs *in planta* is possible to be demonstrated using two immunocytochemical approaches, i.e. immunofluorescence labelling and immunogold labelling (Table 2).

**Table 2:** Application, advantages, and disadvantages of microscopic techniques with antibody-based probes in AGPs studies

Technique	Application	Advantage	Disadvantage
Immunofluorescence labelling—Confocal laser scanning microscope (CLSM)	Used to show where AGPs are distributed across labelled cells and tissues; analyses at the tissue/cellular level	AGP location is identified using a fluorescent signal It is possible to see cellular features at a resolution of 1 µm High contrast and resolution images are possible to obtain quickly and non-intrusively Eliminates the problem of glare resulting from preparation of layers lying outside the plane of focus	The fluorescence lessens with time. The fluorescence of samples fades (photobleaching) during observations Results are susceptible to the effects of the environment Resolution is still inferior to that of electron microscopy A high price and a very limited field of view
Immunogold labelling—Transmission electron microscope (TEM)	Used to localise AGPs and other cell wall components at the subcellular level	AGP localisation in ultra-thin sections using a gold-conjugated antibody It is possible to see intracellular features at a resolution of 0.2 nm Analysis and observation of very high-resolution images High magnification	Fixation is needed in cell preparation, which might result in artificial damage Lengthy and complicated methods for preparation of cells and tissues for TEM Requires a conductive coating of gold/palladium alloy, carbon, osmium, etc. for correct imaging Expensive, difficult to access, and complicated to use

#### Protocol of tissue preparation for microscopic methods

**Fig. 1:**
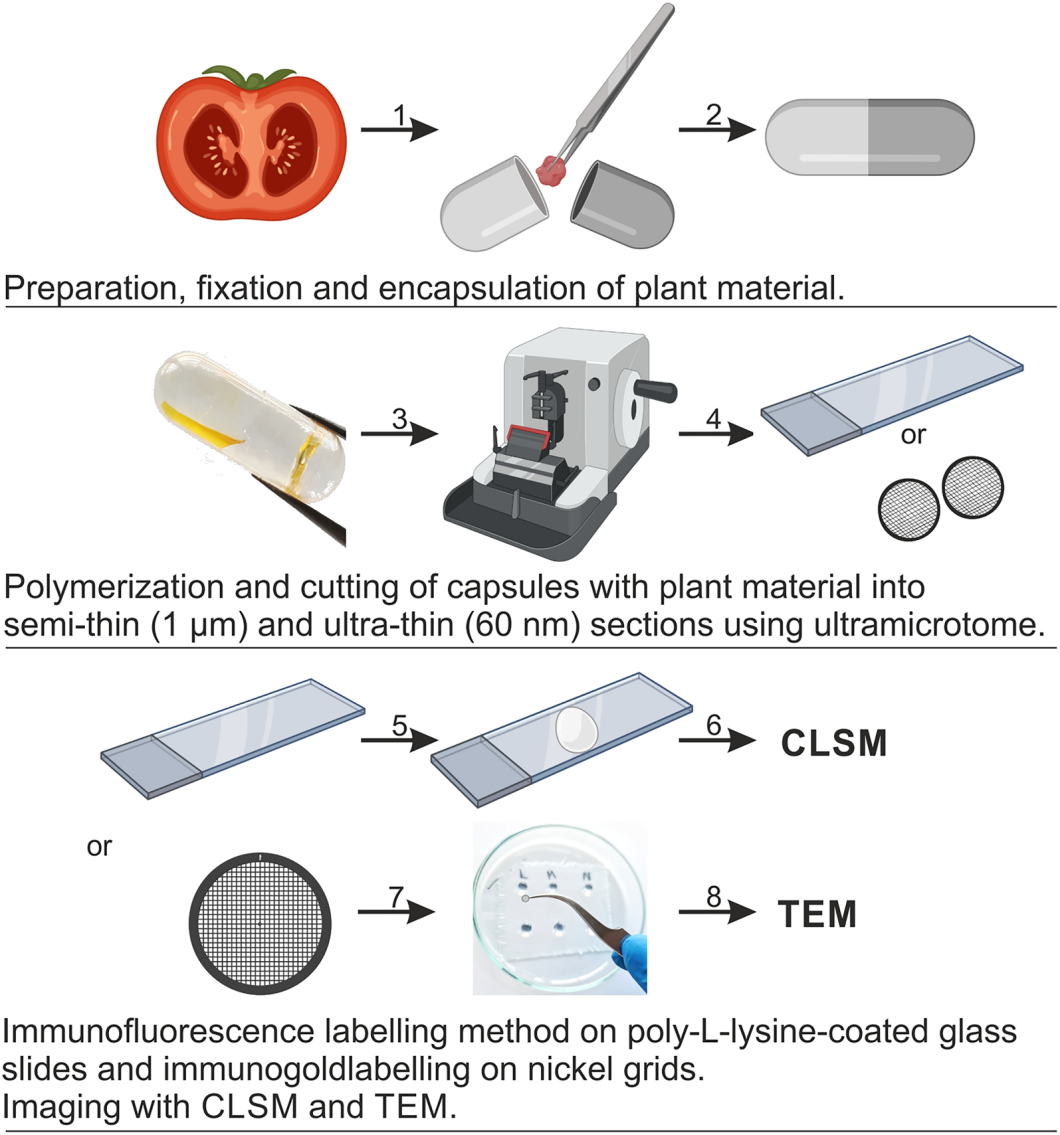
Scheme of the preparative sequence of microscopic methods. (1) Preparation, fixation, and encapsulation of plant material. (2) Polymerisation of capsules with fixed plant material. (3) Cutting capsules into semi-thin and ultra-thin sections. (4) Mounting the sections on poly-L-lysine-coated glass slices (for CLSM) or nickel grids (for TEM). (5) Immunofluorescence labelling – method on poly-l-lysine-coated slices. (6) Imaging with CLSM. (7) Immunogold labelling – method on grids with formvar film (8) Imaging with TEM. Created with BioRender.com

The tissue of fresh fruit is very delicate, highly hydrated, and prone to damage. Hence, preparation of sufficient thin sections with appropriate preparative steps is typically advantageous. The material should be subjected to the procedure of fixation, resin embedding, and thin or/and ultra-thin sectioning [42, 43]. The basic steps of sample preparation for CLSM and TEM are the same: fixation in a fixative solution, dehydration in gradient series of ethanol solutions, embedding in resin, and polymerisation. The choice of chemical fixative and buffer solutions depends on the purpose of the CLSM and TEM study and requires optimisation of the procedure for fruit tissue for both structural studies and labelling of AGP carbohydrate epitopes. A common problem encountered during the fixation step is the use of glutaraldehyde. Also, the embedding stage and the use of proper resin are fundamental to the final quality of sample blocks and sectioning. Epoxy resins cannot be used for CLSM and TEM imaging of fruit tissues compared with other plant tissues [42, 43]. This is due to its high viscosity and potential to damage cellular structures. Another disadvantage of this resin is its poor staining ability, which is a basic criterion for microscopic analysis of fruit tissues. LR White resin is a low-viscosity and non-toxic acrylic resin with minimal non-specific staining, which makes it an ideal tool for infiltrating fruit tissues. Moreover, LR White resin provides a chance of using one block for both CLSM and TEM, which definitely advances the sectioning step. Also, sections of LR White resin are hydrophilic so that immunocytochemistry reagents can easily penetrate into the section. The scheme of the method is shown in Fig. 1.

##### Procedure

1. Cut cube-shaped pieces of fruit tissue.
2. Add 2% paraformaldehyde and 2.5% glutaraldehyde in PBS (phosphate-buffered saline) to the fruit tissue.
3. Place the tissue with the fixation solution in a vacuum (0.7 bar) seven times, with breaks after vacuum for 10 min each. Next, place the tissue under vacuum overnight.
4. Wash the sample with PBS three times for 15 min each at RT (room temperature).
5. Remove the solution and wash the sample with distilled water three times for 15 min each at RT.
6. Remove the solution and start the dehydration process with graded series of ethanol solutions. Add 30%, 50%, 70%, 90%, and 96% ethanol for 15 min each at RT.
7. Remove the solution and add 99.8% ethanol twice for 30 min each at RT.
8. Substitute ethanol with 3:1, 1:1, and 1:3 mixtures of 99.8% ethanol and LR White resin for 2 h each at RT and with 100% LR White resin overnight at 4 °C.
9. Remove the solution and replace by 100% LR White resin (infiltration of the sample with LR White resin). Leave the sample for 8 h at RT.
10. Encapsulate the sample in gelatine capsules and start polymerisation for 48 h at 55 °C.
11. Prepare a cutting plane of an appropriate size on blocks containing the embedded material (trimming stage).
12. Cut semi-thin Sects. (1 µm) using a glass knife-equipped ultramicrotome. Mount the sections on poly-l-lysine coated glass slides.
13. Cut ultra-thin Sects. (70 nm) using a diamond knife-equipped ultramicrotome. Mount the sections on formvar film-coated nickel square grids.

##### Critical

1. Samples should not be left in 100% resin at RT longer than a few days; otherwise, they will begin to polymerise.
2. Using LR white to place fruit tissue requires a graded series of ethanol, not acetone, because acetone residues may hinder polymerisation.
3. The steps of tissue saturation with resin cannot be accelerated, because insufficient time of this step will result in poor fixation of the tissue, which will make it impossible to section it. The block inside will be too soft.
4. Nickel square mesh grids with formvar, not the more common copper grids, should be used to place the sections for electron microscopy, as they are non-reactive and do not react with antibodies.

#### Immunofluorescence labelling with antibody-based probes and CLSM imaging at the cellular level

**Fig. 2:**
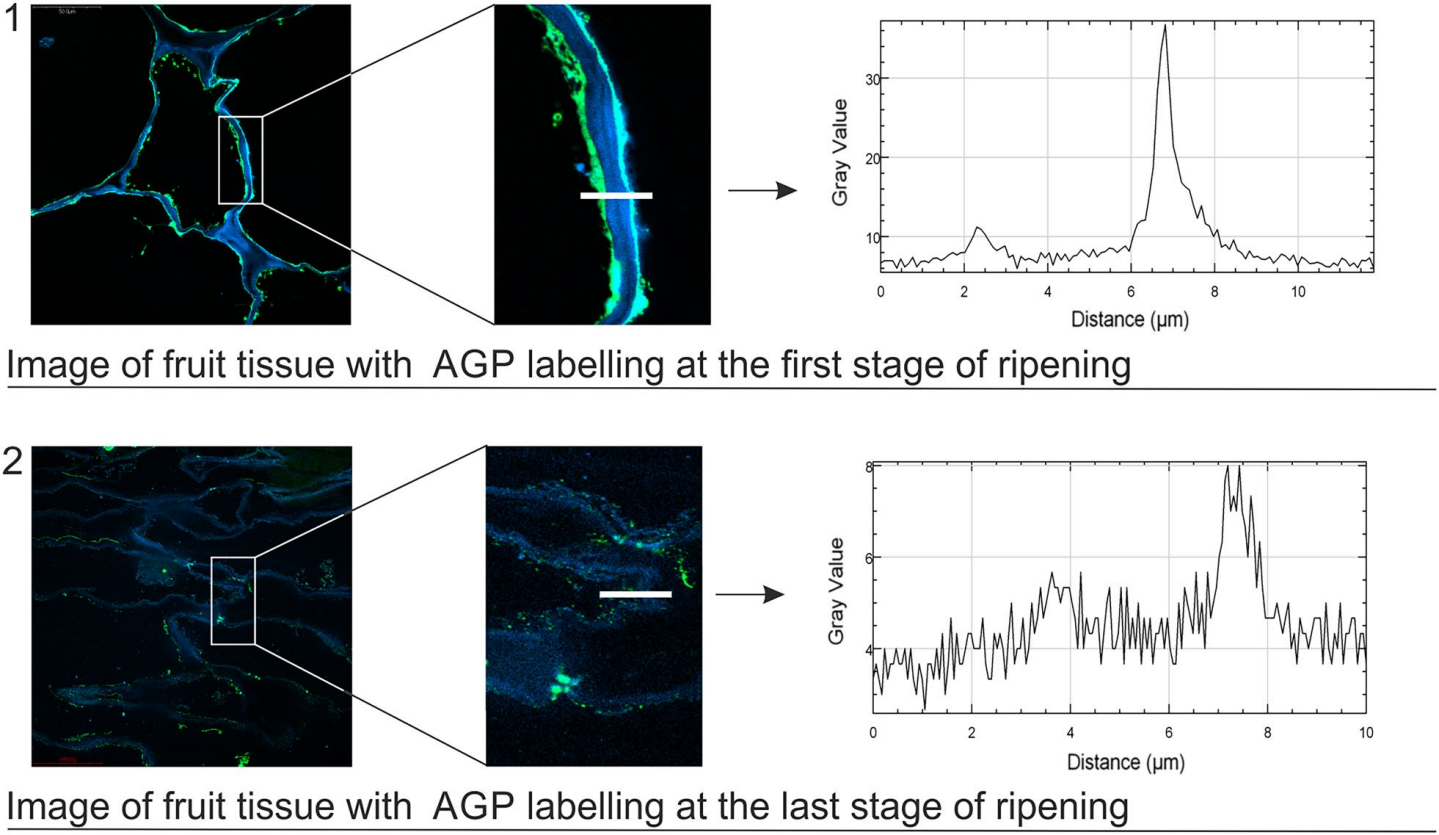
CLSM image with a magnification of the cell wall in fruits at the first stage of ripening (1) and at the last stage of ripening (2). Results obtained with the immunofluorescence method – plots of the grey value profile from labelled cell walls (marked with a white line)

Understanding the precise distribution of AGPs in fruit tissues is of paramount importance because of their pivotal roles in fruit growth, development, and ripening. Immunofluorescence enables researchers to visualise AGPs within fruit tissues, thereby offering insights into their quantity, localisation, dynamics, and interactions with other cellular components [44, 45]. In the immunofluorescence technique, fruit sections are placed on poly-l-lysine-coated slices, which are incubated with primary monoclonal antibodies that recognise AGP carbohydrate moieties (Table 1). To label fruit tissue, we employed antibodies that were more diluted than those typically used in other studies and as recommended by the producer. This 1:10 concentration proved to be too high, resulting in nonspecific binding and poor visualization of the AGP epitopes' distribution [34]. Subsequently, to visualise AGP epitopes, the sections are incubated with secondary antibodies conjugated with a fluorescent label [6, 44, 46]. The intensity of fluorescent signals is determined from CLSM images, which reveals the quantity of AGP epitopes in the sample [44, 47, 48]. Due to the distribution of AGPs at the border of the cell wall and membrane compartments, it is necessary to use Calcofluor White Stain for better visualisation of the AGP epitopes. This fluorescent blue dye staining cellulose allows labelling of the whole surface of the cell wall. In the case of samples from various stages of ripening, quick disappearance of immunofluorescence can be observed at the last stages of the process, which is correlated with the lower content of AGPs and the lower number of epitopes. Dako Fluorescent Mounting Medium, which retards fading of the fluorescence signal and thus allows longer imaging after mounting, can be used to enhance the visualisation of fluorescence. The schematic description of the method is shown in Fig. 2.

##### Procedure

1. Prepare semi-thin (1 µm) sections using an ultramicrotome and a glass knife.
2. Place the sections on poly-l-lysine coated glass slides and dry at 45 °C.
3. Use a liquid blocker Dako-Pen to draw a hydrophobic barrier around the sections on the glass [49].
4. Wash the slice with PBS twice for 15 min each.
5. Add blocking buffer (2% BSA solution in PBS) and incubate for 30 min at RT.
6. Remove the blocking buffer and wash the slice with PBS for 15 min at RT.
7. Add primary antibody diluted at 1:50 in a 0.1% BSA solution in PBS and incubate overnight at 4 °C. Next day, incubate for 15 min at RT.
8. Remove the solution and wash the slice four times with PBS for 20 min each at RT.
9. Add secondary antibody conjugated with a fluorescent label diluted at 1:200 in a 0.1% BSA solution in PBS and incubate overnight at 4 °C. Next day, incubate for 15 min at RT.
10. Remove the solution and wash the slice with PBS twice for 15 min each at RT.
11. Wash the slice five times with distilled water for 10 min each at RT.
12. Stain the slice with Calcofluor White in order to contrast the image.
13. Visualise the sections using CLSM.
14. Make control reactions without incubation with the primary antibody.

##### Critical

1. It is important to place sections onto poly-L-lysine-coated glass slides to prevent their run-off during the next immunolabelling steps.
2. The drying of slides with sections cannot be accelerated by raising the temperature above 45 °C. This causes rippling of polymerised tissue, which reduces the quality of immunofluorescence labelling and CLSM imaging.
3. The entire labelling procedure must take place in a wet enclosed space.
4. A few additional solutions can reduce unspecific binding: adding the detergent Tween to the wash buffer, using excess volumes of wash buffer, and trying to remove all wash buffer by tapping the glass against absorbent paper.
5. The step with secondary antibody, i.e. Alexa-Fluor 488, should be carried out in the dark.

#### Immunogold labelling and imaging at the subcellular level using a TEM

**Fig. 3:**
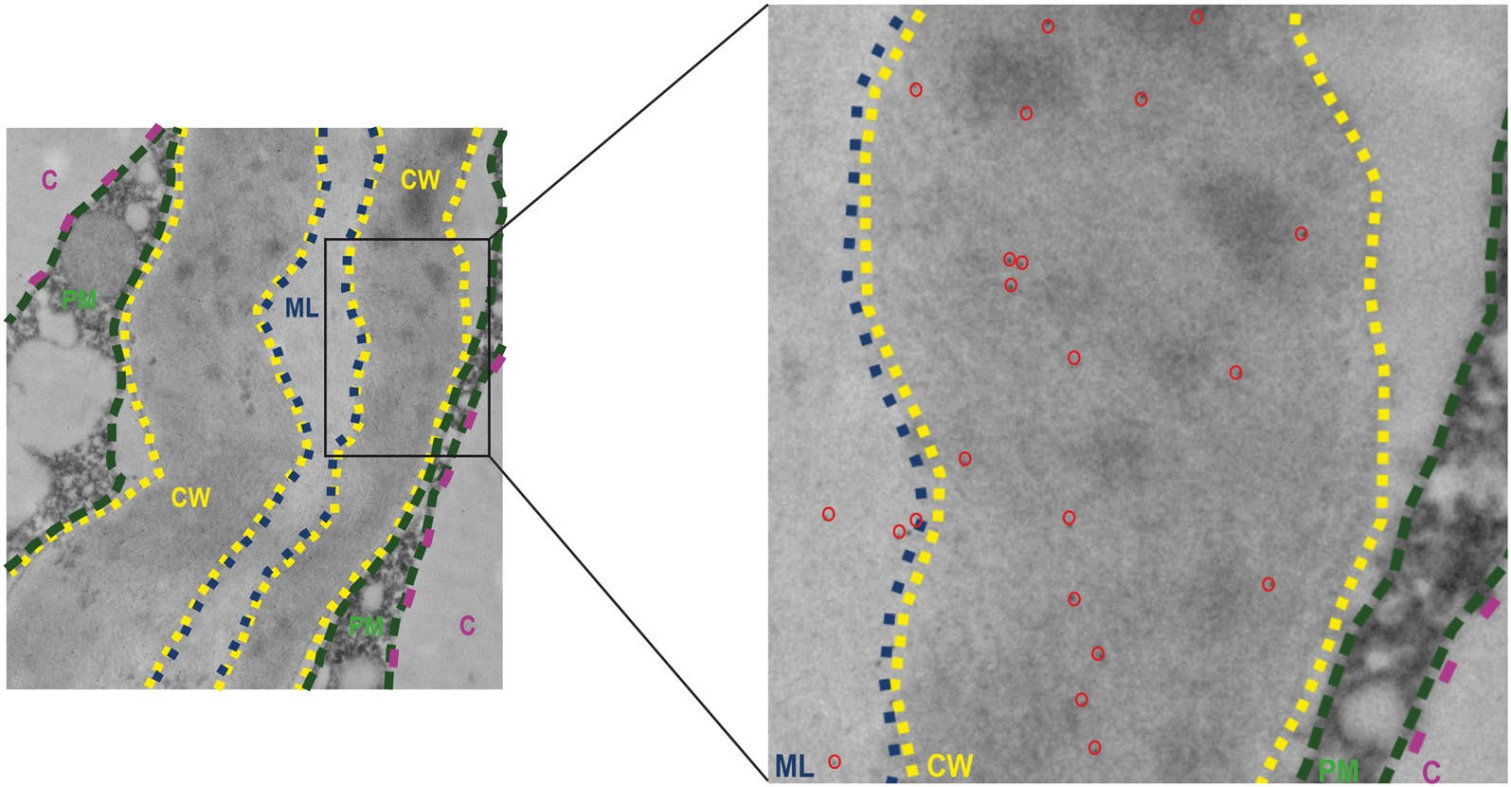
TEM image with labelled AGPs. Epitopes are visible as dark dots (circled in red colour). Magnification with underlined localisation of AGPs in particular cell compartments. Abbreviations: CW – cell wall (yellow colour), PM – plasma membrane (green colour), ML – middle lamella (blue colour), C – cytoplasm (pink colour)

The immunogold labelling method, which merges the resolution of electron microscopy with the specificity of immunolabelling, allows for the precise visualization of AGPs at a subcellular level of fruit tissue. By using secondary antibodies conjugated with gold nanoparticles, AGPs can be identified and allowed to reveal their involvement in the cell wall assembly, signalling, and intercellular communication [40, 43]. Like the other immunocytochemical methods, immunogold labelling used for AGP studies require technical modifications in the tissue preparation protocol [50]. Using primary monoclonal antibodies that target particular AGP epitopes, fruit sections are placed on grids covered by a formvar layer [51]. An antibody conjugated with colloidal gold particles specific to the primary antibodies should be used as a secondary antibody. Due to their high electron density and punctate shape, coupled colloidal gold particles are simple to identify as dark dots and count. In contrast to the standard plant sample preparation protocols [50], the osmium tetroxide treatment is excluded. This step is omitted to avoid worsening the quality of the samples, as the use of OsO4-treated often results in a decrease in the intensity of labelled dots during TEM imaging of fruit tissues. Nonetheless, the details within the cells are still well preserved without this solution. The schematic description of the method is shown in Fig. 3.

##### Procedure

1. Prepare ultra-thin (70 nm) sections using an ultramicrotome and a diamond knife with a 45° angle.
2. Place the sections on formvar film-coated nickel grids.
3. Place parafilm on a Petri dish which is necessary to make immunogold labelling.
4. Wash the grids with distilled water three times for 10 min each.
5. Add blocking buffer (1% BSA solution in PBS) and incubate for 30 min at RT.
6. Add primary antibody diluted at 1:10 in a 0.1% BSA solution in PBS and incubate for 3 h at 37 °C.
7. Remove the solution and wash the grids with a 1% BSA solution in PBS three times for 10 min each.
8. Add secondary antibody conjugated to gold particles diluted at 1:50 in a 0.1% BSA solution in PBS and incubate for 1 h at 37 °C.
9. Remove the solution and wash the grids with PBS twice for 10 min each.
10. Wash the grids with distilled water five times for 10 min each.
11. Allow the grids to dry on clean filter paper and next put them into the TEM Grid storage box.
12. Add a filtered aqueous 1% UA solution (uranyl acetate) and incubate for 10 min at RT.
13. Wash the grids with distilled water three times for a few seconds each and allow them to dry.
14. Add filtered Reynold’s reagent (triple lead citrate) and incubate for 7 min at RT.
15. Wash the grids three times with distilled water for a few seconds each, allow them to dry, and transfer them to the grid box for storage.
16. Visualise sections and examine the image using TEM.
17. Make control reactions without incubation with the primary antibody.

##### Critical

1. Filter both staining solutions using a 0.2 µm filter, as this step will prevent contamination of the grids.
2. The grid must be floating, not submerged.
3. To prevent grid contamination, place NaOH pellets around the staining vessel to absorb CO_2_ and wetness.

### Ex situ studies – molecular methods

Ex situ methods for molecular analysis are an ideal way to analyse AGPs isolated from fruit tissue. These methods are based on investigations of material obtained in the extraction procedure, which allows qualitative and quantitative analyses. Modern molecular methods not only detect the presence of AGPs but also determine their concentration and structural characteristics [52–54]. The ability to demonstrate the presence and amount of AGPs in the sample is possible using two molecular techniques, i.e. Western blotting and ELISA test [55] (Table 3).

**Table 3:** Application, advantages, and disadvantages of molecular techniques with antibody-based probes in AGPs studies

Technique	Application	Advantage	Disadvantage
Western blotting	Used to detect the molecular weight of AGPs	Separation of proteins according to molecular weight High specificity and sensitivity Nice and clean interpretable results Commercially available antibodies	Work-intensive and not quick Medium throughput High cost and technical demand Tissue must be homogenised Low intensity of bands can distort the results
ELISA	Used to detect AGPs qualitatively and quantitatively	High specificity, sensitivity, and efficiency Easy to perform with a simple procedure Commercially available reagents for detection Simultaneous analysis of several samples	High cost Insufficient blocking of immobilised antigens yields false results Results limited to the amount of the antigen in the sample

#### Tissue preparation—extraction protocol

Generally, the extraction of AGPs from plant tissue is complicated because the sugar residues of AGPs are linked to other cell wall constituents, which additionally contributes to cell wall stability and resistance to chemical, physical, and biological factors. Therefore, the extraction process must be aggressive enough to access AGPs without irreversible alterations in the protein moiety. Compared to more rigid plant structures, such as stems or leaves, fruit tissue is more hydrated and changes its biochemical composition throughout physiological processes. These factors make the extraction process more complex, and the concentration of the extraction buffer during AGPs extraction should be considered. Before the extraction process, the fruit tissue is sliced and frozen at − 80 °C. In order to extract proteins from plant tissues, they must be initially homogenised in liquid nitrogen and then added to suitable extraction buffer (i.e. Laemmli’s buffer) [42, 56]. Modified Laemmli’s buffer is composed of 65 mM Tris–HCl pH 6.8 (tris-(hydroxymethyl)-aminomethane), 2% SDS (sodium dodecyl sulphate), 2 mM EDTA (ethylenediaminetetraacetic acid), 1 mM PMSF (phenylmethylsulphonyl fluoride), 700 mM β-mercaptoethanol, and 1:10 protease inhibitor. This extraction buffer is used to maintain the pH, ionic strength, and stability of AGPs during the extraction procedure. For a less hydrated fruit tissue, a proportion of 1:1 should be used (i.e. 1 mL of extraction buffer per 1 g of tissue). This proportion should be modified (i.e. 0.5 mL of extraction buffer per 1 g of tissue) in the case of a highly hydrated fruit tissue from the last stages of ripening. In order to prevent protein degradation, samples should be kept on ice during the extraction process and extracted quickly with pre-chilled equipment. The homogenates are typically boiled for 5 min at 95 °C and clarified by centrifugation at 14 000 rpm at 4 °C for 20 min. The last step of the extraction process is to collect the supernatant, which is ready for use or/and may be frozen at −80 °C.

#### Molecular mass characterisation of AGPs using immunoblotting

**Fig. 4:**
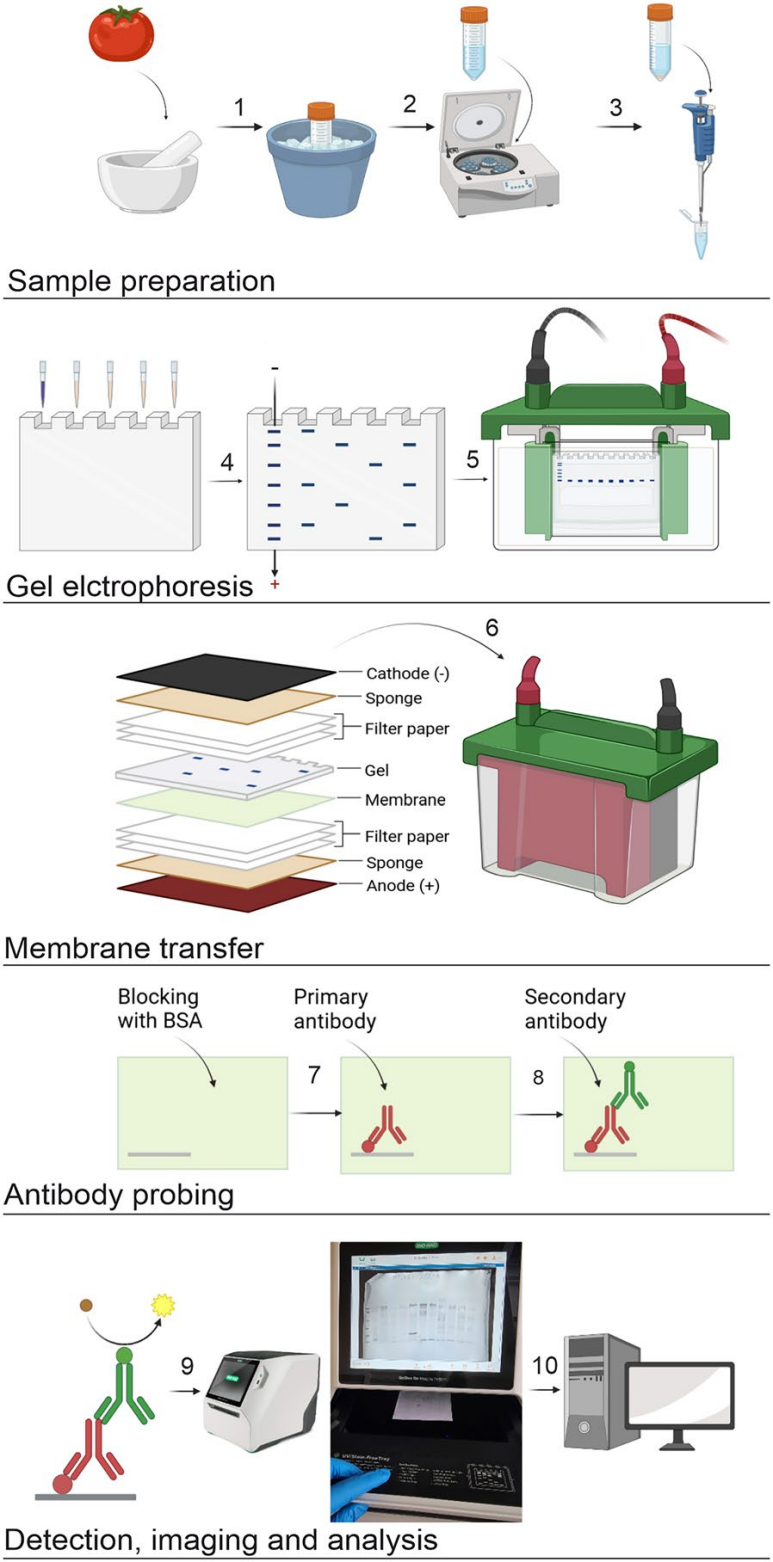
Scheme of measuring AGPs using Western Blotting. (1) Homogenisation of plant material in liquid nitrogen. (2) Extraction step. (3) Collection of the supernatant. (4) Application of samples and protein leader to electrophoresis gel. (5) SDS-PAGE electrophoretic separation. (6) Preparation of the sandwich. Membrane transfer. (7) Membrane blocking with BSA and incubation with primary antibody. (8) Membrane incubation with secondary antibody. (9) Signal detection and band measurement. (10) Imaging with a scanning machine. Created with BioRender.com

Immunoblotting enables specific detection and molecular mass characterisation of AGPs in fruit tissues, which allows the demonstration of their structural variations and functional diversity. Western blotting is an analytical technique used in molecular biology to detect and characterise specific antigenic determinants [54, 57, 58]. The technique involves appropriate sample preparation, electrophoretic separation (i.e. SDS-PAGE – sodium dodecyl sulphate polyacrylamide gel electrophoresis) [7, 59, 60], transfer of separated proteins from the gel to the membrane (nitrocellulose, polyvinylidene difluoride (PVDF)) [61, 62], incubation with appropriate antibodies, and detection [63]. Visualisation of these markers can be carried out colorimetrically or with chemiluminescence methods [53, 57]. To optimise the Western blotting technique used to study AGPs, some changes should be done in the concentrations of the reagents used [64]. First of all, the AGP epitope analysis of fruit tissues requires the optimal concentration of the antibody, which is determined by testing a variety of antibody dilutions around the concentration suggested by the producer. To improve the imaging AGP epitopes on the membrane, lower concentrations of antibodies were used than the 1:10 dilution typically used for immunoblotting plant samples, such as from *Brassica napus* (var. Expert) and pea (*Pisum sativum* var. Normand) roots [63]. Also, to improve imaging and minimise a high background, the concentration of the blocking buffer should be modified due to the necessity of more effective blocking of non-specific sites. Additionally, in AGP separation, a wet membrane transfer at 4 °C should be carried out to minimise unwanted effects of generated heat, such as gel distortion. The schematic description of the method is shown in Fig. 4.

##### Procedure

1. To construct polyacrylamide gel electrophoresis with protein denaturing conditions (SDS-PAGE gel), prepare the resolving gel: mix 6 mL of 12.5% resolving gel (stock: mix 33.28 mL of acrylamide, 25.12 mL of distilled water, 20 mL of 1.5 M Tris pH 8.8, 800 µL of SDS), 60 µL of 10% APS (ammonium persulphate sodium), and 20 µL of TEMED (N,N,N',N'-tetramethylethylene diamine), cast it between two glass plates, pour over ¾ of the volume, and wait for polymerisation for about 30 min. After polymerisation, add 2 mL of isopropanol to leave a flat surface of the resolving gel.
2. Remove the alcohol by touching with the edge of tissue paper. Prepare the stacking gel: mix 3 mL of the stacking gel (stock: mix 5.44 mL of acrylamide, 21.76 mL of distilled water, 4 mL of 1 M Tris pH 6.8, 320 µL of SDS, Bromophenol blue), 30 µL of 10% APS and 15 µL TEMED, cast it between two glass plates, insert a plastic electrophoresis comb to create the wells, and wait for polymerisation for 30 min.
3. Prepare samples: add 3 × SDS sample buffer to the total protein amount (according to Bradford’s measurement), mix the samples, and heat the mixture to 95 °C for 5 min.
4. Set up the electrophoresis apparatus and add 1 × running buffer (mix 14.4 g of glycine, 3 g of Tris Base, 1 g of SDS, and 1 L of distilled water).
5. Load an appropriate protein ladder and samples onto the gel (around 20 µL per well).
6. Turn on the electrophoresis power pack and set it to a low voltage (80 V for 20 min) with a subsequent increase to a higher voltage (120 V for 1 h). Stop the gel running when the protein leader migrates to the appropriate position.
7. Wash PVDF membranes (nitrocellulose membranes) in methanol for 1 min before transfer.
8. Soak sponges and Whatman filter paper in 1 × transfer buffer (mix 14.41 g of glycine, 15.14 g of Tris Base, and 4 mL of 10% SDS in 100 mL of distilled water and add 200 mL of methanol and 700 mL of distilled water).
9. Assemble the transfer constituent ‘sandwich’ sequentially arranging the sponge, 2–3 sheets of wetted filter paper, the membrane, the gel, 2–3 sheets of wetted filter paper, and finally the second sponge, starting to build on the transfer cassette facing the anode ( +).
10. Turn on the power pack, set the voltage to low, and use the wet transfer technique (90 V for 80 min).
11. Wash the membrane with TBST (Tris-buffered saline) on a lab shaker for 15 min.
12. Add blocking buffer (5% BSA solution in PBS) and incubate for 1 h at RT on a lab shaker.
13. Remove the blocking buffer and wash the membrane with TBST three times for 5 min each on a lab shaker.
14. After the preincubation step, add the primary antibody diluted in 1:500 in a 2.5% BSA solution in PBS and incubate for 2 h at RT on a lab shaker or overnight at 4 °C.
15. Remove the solution and wash the membrane with TBST three times for 5 min each on a lab shaker.
16. Add secondary antibody with the AP-specific (alkaline phosphatase) enzyme conjugate diluted at 1:1000 in a 2.5% BSA solution in PBS and incubate the mixture for 2 h at RT on a lab shaker.
17. Remove the solution and wash the membrane with TBST three times for 5 min each on a lab shaker.
18. Wash the membrane for 10 min with the AP buffer (mix 12.11 g of Tris Base, 5.84 g of NaCl, 1.01 g of MgCl2, and 1 L of distilled water, pH 9.5).
19. Add a freshly prepared substrate solution for colorimetric detection (20 mL of AP buffer, 1 mL of BCiP (5-bromo-4-chloro-3-indolyl phosphate), and 1 mL of NBT (nitrotetrazolium Blue chloride) to the membrane.
20. Incubate the membrane at RT in the dark on a lab shaker until bands are reached (the average time is 15 min).
21. Wash the membrane twice with distilled water for 10 min each.
22. Membrane imaging using an imaging system with a UV free tray.

##### Critical

1. To prevent degradation of AGPs, samples should be kept on ice during preparation and application on the gel.
2. Prepare and store BCiP and NBT reagents in the dark.
3. If a too high background is noted on the membrane, it requires shorter incubation with primary antibodies.
4. If “smiling” bands are observed, the SDS-PAGE electrophoresis was overheated or ran too quickly; in such a case, either the voltage should be lower or the electrophoresis should be run in colder conditions.
5. Any white spots noted on the membrane suggest that probably air bubbles were left between the gel and the membrane during the folding of the ‘sandwich’; to resolve this, moisten the plastic with transfer buffer and use it to roll air bubbles out of the membrane.
6. Any black spots noted on the membrane suggest that probably the antibodies bound to the blocking reagent; to resolve this, use a different blocking reagent and wash the membrane more precisely before detection.
7. AGPs (in comparison to typical proteins) do not separate as single bands but as smeared bands. This is a result of the heterogeneity of glycosylation.

#### Selective glycome profiling of AGPs using enzyme-linked immunosorbent assay—ELISA test

**Fig. 5:**
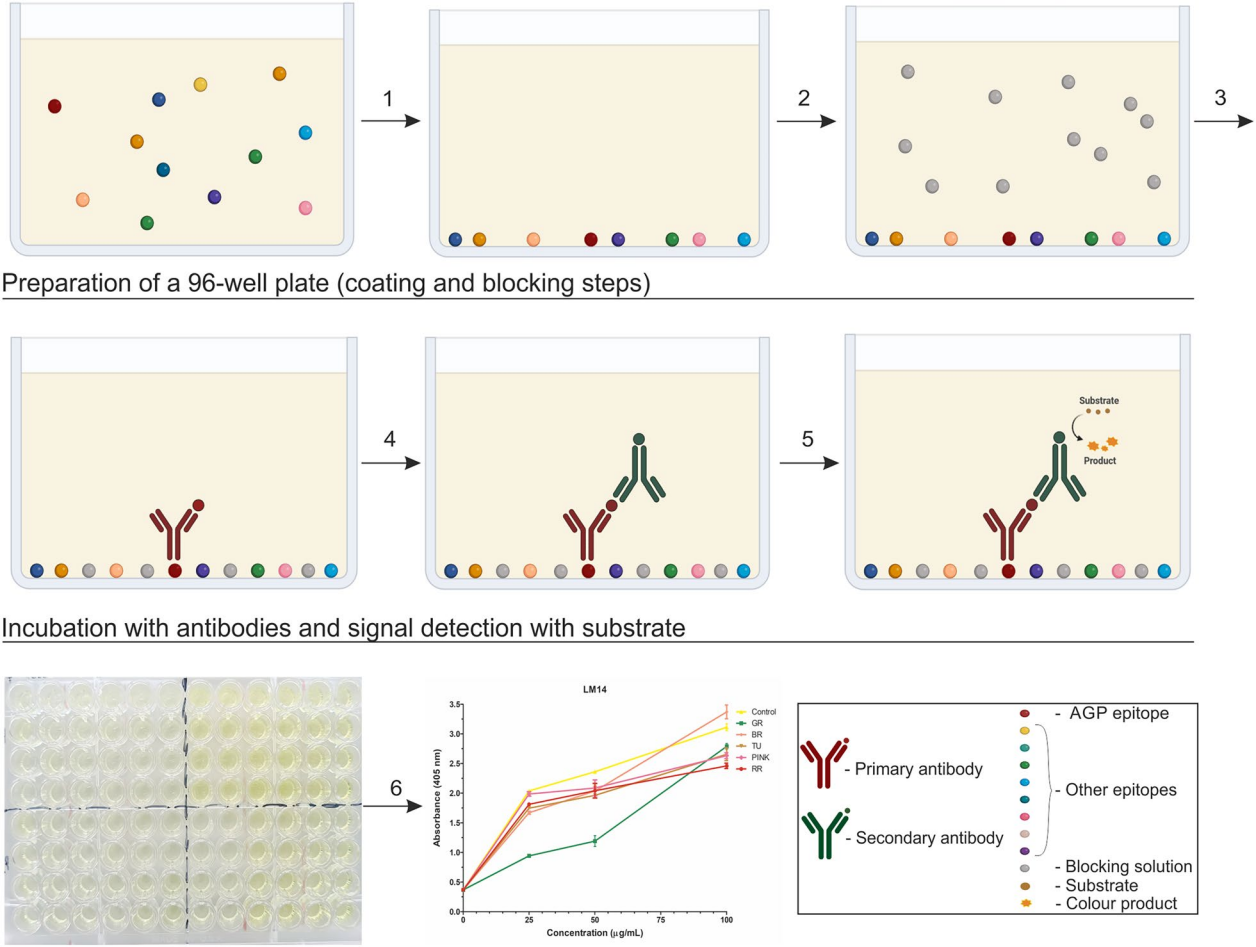
Scheme of measuring AGPs using ELISA. (1) Coating – sample immobilisation. (2) Blocking with BSA. (3) Incubation with primary antibody. (4) Incubation with secondary antibody. (5) Signal detection using a microplate reader. (6) Measurement and quantitative analysis of the AGP concentration in the sample. Created with BioRender.com

ELISA is an immunoenzymatic test commonly that is used in scientific research. Its advantages include high sensitivity [65] and the ability to evaluate multiple samples at the same time providing highly reproducible results [52, 66]. ELISA is a basic test for the qualitative and quantitative determination of specific epitopes based on the number of antigen–antibody bonds formed. By comparing these bonds to standards, it is possible to estimate their quantity [67, 68]. AGPs can also be analysed qualitatively and quantitatively using ELISA. However, due to the specific structure of AGPs, modification of the protocol is necessary. In the case of fruit tissue, which are highly hydrated, the cover process is extended to 72 h, as opposed to a few hours in traditional ELISA. Additionally, it is run at 37 °C, compared with overnight coating at 4 °C for rice and carrot roots [33]. These modifications have made it possible to optimise the sample covering. Moreover, optimisation of the concentration of the primary antibody was carried out, as the use of the recommended concentration of 1:10 by the producer gave too intense a background signal [25, 34]. The description of the method is shown in Fig. 5.

##### Procedure

1. Prepare a 96-well microplate and add particular sample per well.
2. Incubate for 72 h at 37 °C with shaking (350 rpm).
3. Wash the plate with 100 μL of PBS three times for 5 min each on a lab shaker.
4. Add 200 µL of blocking buffer (0.1% BSA solution in PBS) per well and incubate for 1 h at 37 °C with shaking (350 rpm).
5. Remove the blocking buffer and wash the plate with 100 μL of PBS three times for 5 min each on a lab shaker.
6. After the preincubation step, add 100 μL of primary antibody diluted at 1:20 in PBS per well.
7. Cover the plate and incubate for 1 h at 37 °C with shaking (350 rpm).
8. Remove the solution and wash the plate with 100 μL of PBS three times for 5 min each on a lab shaker.
9. Add 100 μL of secondary antibody with the enzyme conjugate an AP-specific diluted at 1:500 in PBS per well.
10. Cover the plate and incubate for 1 h at 37 °C with shaking (350 rpm).
11. Remove the solution and wash the plate with 100 μL of PBS three times for 5 min each on a lab shaker.
12. Add 100 μL of a freshly prepared substrate solution of PNPP (p-nitrophenol phosphate) per well.
13. Incubate the plate at RT in the dark until the desired colour intensity is reached (on average it is 15 min).
14. Add 50 µL of 2 M NaOH to stop the reaction.
15. Measure the absorbance at 405 nm.

##### Critical

1. Equilibrate PNPP to room temperature and keep it in the dark before using.
2. Too strong a signal (no differentiation on the board) most probably indicates that the wrong concentration of antibody or too much substrate was used or the plate was not rinsed properly between the steps.

Briefly, all specific reagents, buffers, and types of equipment that are necessary for immunocytochemical analyses of AGPs are summarised in Table 4. Also, the optimised concentration of primary and secondary antibodies is shown as an integral part of all described methods.

**Table 4:** Reagents and equipment necessary for immunocytochemical techniques

Immunofluorescence labelling	Immunogold labelling	SDS-PAGE Western blotting	ELISA
*Reagents*			
PBS	PBS	PBS	PBS
BSA	BSA	BSA	BSA
Paraformaldehyde	Uranyl acetate	12.5% Resolving gel*	NaOH
Glutaraldehyde	Reynolds reagent	Stacking gel*	PNPP
NaOH	Paraformaldehyde	10% APS	
Ethanol	Glutaraldehyde	Sample buffer*	
Resin	NaOH	Running buffer*	
	Ethanol	Transfer buffer*	
	Resin	TBST*	
		AP buffer*	
		BCiP*	
		NBT*	
		DMF	
*Antibodies*			
Primary antibody diluted in BSA (1:50)	Primary antibody diluted in BSA (1:10)	Primary antibody diluted in PBS (1:500)	Primary antibody diluted in PBS (1:20)
Secondary antibody conjugated with a fluorochrome diluted in BSA (1:200)	Secondary antibody conjugated with gold nanoparticles diluted in BSA (1:50)	Secondary antibody with AP diluted in PBS (1:1000)	Secondary antibody with AP diluted in PBS (1:500)
*Equipment*			
Vacuum pump	Vacuum pump	Electrophoresis apparatus	Microplate washer
Gelatine capsules	Gelatine capsules	Wet transfer module	Microplate shaker
Ultramicrotome	Ultramicrotome	Laboratory shaker	Microplate reader
Glass knife	Diamond knife	Nitrocellulose membrane	
Poly-L-lysine coated glass slides	Nickel grids with formvar film	Scanning machine	
Wet chamber	Petri dishes		
Hydrophobic pen	TEM		
CLSM			

^*^Resolving gel—12.5% (33.28 mL of acrylamide, 25.12 mL of distilled water, 20 mL of 1.5 M Tris pH 8.8, 0.8 mL of 10% SDS)

^*^Stacking gel (5.44 mL of acrylamide, 21.76 mL of distilled water, 4 mL of 1 M Tris pH 6.8, 0.32 mL of 10% SDS, bromophenol blue)

^*^Sample buffer (100 µL of Tris pH 6.8, 480 µL of 10% SDS, 480 µL of 0.5 M DTT, 240 µL of 100% glycerol)

^*^Running buffer (add 14.4 g of glycerine, 3 g of Tris Base, and 1 g of SDS to 1 L of distilled water)

^*^Transfer buffer (add 72.06 g of glycine, 15.14 g of Tris Base, and 20 mL of 10% SDS to 500 mL of distilled water)

^*^TBST (add 2.423 g of Tris Base, 8 g of NaCl, and 1 mL of Tween 20 to 1 L of distilled water and establish pH 7.6)

^*^AP buffer (add 12.11 g of Tris Base, 5.84 g of NaCl, and 1.01 g of MgCl_2_ to 1 L of distilled water and establish pH 9.5)

^*^BCiP (add 4 mg of BCiP to 1 mL of distilled water)

^*^NBT (add 9 mg of NBT to 0.3 mL of distilled water and 0.7 mL DMF)

## Discussion

AGPs are classified as a family of proteins present in the cell wall structure [15, 69]. Considering their heterogeneous structure, a few types of AGPs should be distinguished [15]. AGPs identified from the *Arabidopsis* genome based on composition, size, and the presence of different domains fall into four distinct classes, like classical AGPs, lysine-rich AGPs, AG peptides, chimeric fasciclin-like AGPs (FLAs), chimeric plastocyanin (PAGs) and other chimeric AGPs [70]. Although they are composed of 90% of carbohydrate moiety, AGPs are definitely different from other cell wall polysaccharides which constitute up to 90–95% of the cell wall mass [71]. The cell wall polysaccharides include pectin, cellulose, hemicelluloses, and different glycoproteins [34]. Cellulose microfibrils with hemicelluloses such as xyloglucans, xylans and other glucans form a load-bearing matrix. In this matrix, spaces between cellulose skeleton are filled with amorphic gel, composed mainly of pectic constituents, like the most common homogalacturonan (HG), rhamnogalacturonan type I (RGI), rhamnogalacturonan type II (RGII) and xylogalacturonans (XG) [69, 71]. The methods listed in current paper with exquisite specificity of monoclonal antibodies have established some aspects and fine details of cell wall structural modifications and distribution of cell wall components during cell metabolism. The structure, presence and amounts of cell wall components vary greatly, starting from the variable localisation in the extracellular matrix, the change in distribution and the significant structural variability observed during the physiological processes of plants. Another feature that distinguishes the other carbohydrate components from AGPs is a pattern of their molecular changes. As far as AGPs can be considered as a marker of particular stages of fruit development and ripening, pectins are more permanent and their alternations cannot be linked to individual processes in the cell [72–74]. Glyco profiling of the main pectic epitopes using ELISA assay by Posé and coworkers on the tomato and strawberry fruit at unripe and ripe stages have defined differently in epitope quantities during the ripening process [75]. The mentioned information allows the conclusion that AGPs, although composed of carbohydrate moiety, exhibit distinct characteristics, indicating novel cell wall attribute.

Taking into account the alternations in content and distribution of AGPs across the examined plant organs, also the necessity of molecular features examination should be underlined to gain additional insight into changes in the glycosylation process. All mentioned above properties of AGPs in comparison to features of other cell wall constituents allow concluding that AGPs may be involved in plant molecular mechanisms. As is well-known AGPs are present both free in the apoplast where they act as signalling molecules as well as in strict connection with the extracellular matrix, in the close area of the cell wall and plasma membrane. In the literature, the role of AGPs as signalling molecules in molecular interactions of intercellular signalling is connected with their Ca^2+^ binding capacity and presumptive engagement in the release of cell-surface apoplastic calcium [1, 18]. Another, substantial function of AGPs is the co-creating of the assembly of the cell wall by crosslinking with other cell wall glycopolymers and intermolecular formation of calcium bridges [20, 76]. The classical AGPs have been assumed to form complexes with pectins and hemicelluloses, by covalently attaching with RG-I and HG linked to Rha residues in AG polysaccharides and with arabinoxylan attached to Rha residue in the RG-I [20]. Mentioned conjugate isolated from *Arabidopsis* suspension culture media was distinguished by a significant amount of RG-I – AGP fraction, which indeed covalently linkage between RG-I and AGPs for a functional wall structure [76]. The latest reports confirm the role of AGPs in creating cell wall continuity, by physical interaction with the pectic domains RG-I and homogalacturonan [77]. The most recent results indicate that cationic AGP domains serve a chaperoning role by catalyzing boron bridging and RG-II dimerization. The authors assume that RG-II and specific AGPs are involved in guiding cell-wall assembly [77].

The framework of plant cell wall glycans and the maintenance of interactions between them should be also investigated in the fruit context. Results obtained with the aforementioned methods are compatible and allow a comprehensive analysis of AGPs present in fruits. Currently, antibodies recognising carbohydrate chains are the only commercially available tools maximally specific to AGPs [24, 26, 33, 34]. These antibodies are used to identify epitopes that are useful markers of tissue modification during particular stages of the fruit development and ripening process [13]. Despite the strong emphasis on the involvement of AGPs in plant physiological processes, most studies focus only on their structural rather than functional characteristics. To date, AGPs have been investigated in only a few kinds of fruits, e.g. strawberry—*Fragaria* x *ananassa* [78], grape—*Vitis vinifera* [79], tomato—*Solanum lycopersicum* [80], olive—*Olea europaea* L. [81], apple—*Malus domestica* [5, 23, 82], and pear—*Pyrus communis* [4]. Thus, the aim of the current paper was to gather the optimised protocols, troubleshooting, and experience gained from experimental work on AGPs in fruits. So far, most reports of AGPs in fruits have been based on the use of the presented protocols, i.e. the spatio-temporal pattern of distribution, tissue specificity, concentration in fruit tissue, characterisation of the glycan structure, and differences in the carbohydrate moiety structure. Using these protocols, it was possible to determine the effect of AGPs on fruit physiological processes, such as development, ripening, and senescence during postharvest storage [12, 14, 83]. Immunocytochemical analyses in situ with CLSM and TEM imaging have confirmed the altered distribution of AGPs during the ripening process, noted as a typical spatio-temporal pattern. At the beginning of the process, AGP epitopes are found mainly in the plasma membrane, where they are accumulated along the cell wall border. For example, immunolabelling with JIM13 showed the presence of the β-GlcA-(1 → 3)-α-GalA-(1 → 2)-α-Rha epitope in the cell wall-plasma membrane continuum, while their assembly in the mature green and stored fruit tissue was disturbed [5]. The immunogold labelling with JIM13 confirmed the specific arrangement of AGPs in the fruit cell wall. Moreover, the results obtained at the subcellular level using TEM indicated the presence of AGPs in other cellular compartments as well, emphasising that the synthesis of carbohydrate chains and protein moiety takes place in the endoplasmic reticulum [82, 84].

Furthermore, in situ and ex situ immunocytochemical techniques demonstrated a correlation between the level of AGP glycosylation and molecular AGP alterations with advancing cellular processes. Selective glycome profiling of AGPs revealed the decreasing molecular weight and concentration of AGPs with the progress of the ripening process [85]. Similar changes during the grape ripening process were observed by Moore and co-workers [79]. The quantity of AGP epitopes (JIM8, JIM13, and LM14) gradually increased during ripening, which was followed by reduction and release of AGPs into the apoplast [79]. Our research on tomato fruits as a model organism for ripening analyses have revealed that fruits at the breaker stage contain AGPs with the highest molecular weight with miscellaneous carbohydrate chains, while AGPs with low molecular weight (~ 30 kDa) are mainly noted in fruits at the red ripe stage. These low-molecular AGPs may be regarded as a marker of the end of the ripening process in tomato fruits. Also, the JIM13 antibody, which detects the most frequent AGP epitope with a molecular weight range of 120–20 kDa, can be used as a tool to determine the presence or absence of AGPs in plant tissue [85].

On the other hand, the literature provides information about the frequent use of Yariv reagent to isolate AGP, but so far there has been no unambiguous description with which AGP structural moiety it binds [86, 87]. It is acknowledged that a chemical compound known as the β-glucosyl Yariv reagent (1,3,5-tri(p-glycosyloxyphenylazo)-2,4,6-trihydroxybenzene, β-GlcY) is often used as a cytochemical reagent for detection, quantification, and purification of AGPs as well as modification of the molecular activities of AGPs [80, 87]. Also, a previous study showed that Yariv reactivity is not dependent on the peptide component of AGPs. β-GlcY binds to β-1,3-galactan chains which are longer than five residues. Most likely, this is related to the sequential trimming of the AG moieties of AGPs using sets of specific glycoside hydrolases [88]. In our previous work on apple fruit, the AGPs concentration was estimated at 2 080 µg/g of the parenchyma tissue of red fruits. In comparison to the presence of AGPs in tomato fruits, the concentration was similar, indicating that fruit tissue is a rich source of the examined proteoglycans [42]. For example, during the turning stage of the ripening process, the AGP concentration was determined to be 3 110 µg/g of fresh tissue, suggesting that the presence of AGPs is correlated with the ongoing synthesis of long chains of AGP carbohydrates [85]. For comparison, research carried out by Kaur and coworkers have determined the amount of precipitated AGP in *Arabidopsis thaliana* in different organs. The amount of AGP was 150 µg/g of fresh weight rosette leaf, 450 µg/g from the stem, 610 µg/g from the root, and 1000 µg/g from the flower [89]. Other research conducted by Lamport confirmed the presence of 30–300 µg AGPs per fresh weight of tobacco leaf [90]. Precluding the necessity to clarify the role of AGPs in fruit metabolism and considering the content of AGP in other plant organs, research on AGP in fruits is even more useful to obtain research material. Previous studies on AGPs in fruits allow concluding that fruit tissue is a good research material for isolation as well as structural and molecular characterisation of AGPs as crucial cell wall components.

## Conclusions and future perspectives

Immunocytochemical techniques, both in situ and ex situ, offer the opportunity to precisely determine the molecular and structural modifications occurring in fruit tissues during development and ripening processes. Antibody-based methods are the primary tool for studying AGPs. All techniques, i.e. glycome profiling, immunoprinting on a membrane, screening by ELISA, and/or epitope mapping allow characterisation of the carbohydrate moiety of AGPs. Immunocytochemical studies that use antibodies to identify and visualise specific AGP epitopes have their advantages and disadvantages. The advantages of immunocytochemistry include high specificity correlated with qualitative and quantitative analysis. On the other hand, the disadvantage of immunocytochemistry is the sample preparation step associated with difficulties in quantitative analysis and great subjectivity in the interpretation of results. Briefly, microscopic methods using antibody labeling are powerful tools that allow visualisation of AGPs and an understanding of tissue architecture. The immunofluorescence and immunogold methods allow for precise imaging of AGP localisation with high specificity, followed by qualitative and quantitative analysis. Western Blotting has also high specificity and allows for quantitative measurement but also requires several steps, including gel electrophoresis, transfer of proteins to a membrane, and detection with antibodies which can be time-consuming and labor-intensive. Therefore, in parallel, it is worth performing an additional analysis, such as ELISA, which is a method with high sensitivity and specificity. ELISA allows to detection of low levels of the tested molecules in samples, and this is extremely important in the case of hydrated fruit tissue. Moreover, the ELISA technique is scalable, which allows for simultaneous testing of many samples and enables quantitative analysis of the quantity.

The development of new antibodies in future research, including those against the protein domain, will allow a more thorough analysis of changes that occur in AGP molecules, determining the mechanism of AGP synthesis, degradation, and action during physiological processes. Moreover, a thorough study of glycobiology methods to investigate the AGP structure and distribution in cell walls may provide new knowledge for applications in the glycoscience area. Moreover, the latest reports on the construction of an analogue of the Yariv Reagent deserve to be emphasized [91]. The synthesis of an azido analogue of the Yariv reagent which is functionalized with a fluorophore allows to creation of a glycoconjugate that binds AGPs and allows the ability to visualize AGPs using fluorescence microscopy. The new probe for studying AGPs provides an opportunity to carry out more sophisticated imaging of AGP localisation. Moreover, the new way of AGPs visualisation will give the opportunity to compare results provided with immunocytochemistry tools [91].

## Data Availability

Not applicable.

## Abbreviations

AG: Arabinogalactan
AGPs: Arabinogalactan proteins
AP: Alkaline phosphatase
APS: Ammonium persulphate sodium
BCiP: 5-Bromo-4-chloro-3-indolyl-phosphate
BSA: Bovine serum albumin
C: Cytoplasm
CLSM: Confocal laser scanning microscope
CW: Cell wall
EDTA: Ethylenediaminetetraacetic acid
ELISA: Enzyme-linked immunosorbent assay
FITC: Fluorescein isothiocyanate
GalA: d-galacturonic acid
Glc: d-glucose
GlcA: d-glucuronic acid
HRGPs: The hydroxyproline-rich glycoproteins family
Hyp: Hydroxyproline
L-Ara: l-arabinose
L-Fuc: l-fucose
L-Rha: l-rhamnose
M.W.: Molecular weight
mAb: Monoclonal antibody
Man: d-mannose
4-Me-GlcA: 4-o-methyl-glucuronic acid
ML: Middle lamella
NBT: Nitrotetrazolium Blue chloride
P4H: Prolyl 4-hydroxylase
PBS: Phosphate-buffered saline
PM: Plasma membrane
PMSF: Phenylmethylsulphonyl fluoride
PNPP: p-nitrophenyl phosphate
Pro: Proline
PVDF: Polyvinylidene difluoride
RT: Room temperature
SDS: Sodium dodecyl sulphate
SDS-PAGE: Sodium dodecyl sulphate polyacrylamide gel electrophoresis
TBST: Tris-buffered saline
TEM: Transmission electron microscope
TEMED: N,N,N',N'-tetramethylethylene diamine
Tris: Tris-(hydroxymethyl)-aminomethane
UA: Uranyl acetate
Xyl: D-xylose
